# The Roles of Proactive Personality and Career Satisfaction in the Impact of Employer Brand Attributes Congruence on Creativity

**DOI:** 10.3390/bs14070610

**Published:** 2024-07-18

**Authors:** Jiexuan Zhang, Fei Zhu

**Affiliations:** Business School, Central University of Finance and Economics, Xueyuan South Road 39, Haidian District, Beijing 100081, China

**Keywords:** employer brand, congruence, creativity, career satisfaction, proactive personality

## Abstract

Although the relationship between employer brand and employee creativity has become a popular theme, this nexus is indirect and ambiguous. Additionally, most studies ignore the consistency of instrumental and symbolic attributes when discussing the consequences of employer brand. This study explored the mechanism of employer brand attributes congruence on employee creativity through career satisfaction, and further revealed the moderating role of proactive personality. Based on the cue consistency theory and the social information processing theory, a polynomial regression model was created and a response surface analysis was conducted using 488 paired questionnaires. The results showed that employer brand attributes congruence impacted employee creativity via career satisfaction. A consistent employer brand strategy is more effective for the creativity of less proactive individuals, while a high-level proactive personality can compensate for the deficiencies of employer brand attributes incongruence. The results complemented employer brand research from the perspective of the instrumental–symbolic attribute configuration and provided supportive empirical evidence of employer brand practices aiming at improving employee creativity. This study has certain practical implications for HR practitioners.

## 1. Introduction

Innovation plays a crucial role in the transformation of digitization for organizational sustainable development and decisive competitiveness. Previous studies have highlighted the essential role of human resource management practices on employee creativity and organizational innovation [1]. Employer brand (EB) [2] is regarded as an effective human resource management practice in highly market-oriented employment, not only for promoting the quantity and quality of the applicant pool [3,4,5,6], but also for motivating and retaining current employees, further improving organizational performance [3]. Although the relationship between employer brand and employee creativity has become a popular theme recently, some issues remain to be addressed.

First of all, most studies treat employer brand as an overall construct or limit on one of these attributes, which ignores the congruence of the instrumental–symbolic attributes of employer brand [4] and fails to reflect real management practice. Practically, both internal and external employer brands might deliver imbalanced or conflicting signals, manifesting as the discrepancies between two dimensions of employer brand, such as the ignorance of one aspect when investing in another, or the mixture of positive and negative information [5]. Empirically, researchers confirmed the strong correlation and high number of interactions between the two dimensions of employer brand. For example, previous research has confirmed that symbolic and functional attributes interact to predict job seekers’ attraction to the firm [6,7]. Theoretically, based on the cue consistency theory (CCT) [8], individuals rely on multiple external cues to make sense of their surrounding environment, with consistent cues exerting a stronger influence over individuals’ thoughts, feelings, and actions in general [9]. Taken together, we deem it inappropriate to treat the two dimensions as an overall construct or to solely consider one of them when discussing the relationship between employer brand and employee consequences, which might be blamed for the inconsistency in the empirical findings on the effects of employer brand and the lack of robustness of the research [10].

Second, the employer brand–employee behavior nexus is indirect and ambiguous, which means that the black box between their relationship and some boundary conditions needs to be clarified. Previous studies have mostly focused on the effectiveness of employer brand on organizational image, organizational attractiveness, and the quantity and quality of applicants in the recruitment stage [11,12,13]. Nonetheless, the significance of employer brand is also reflected in post-employment. In other words, organizations should not only strive to recruit talent but also contemplate the strategy to motivate their proactivity to enhance the organization’s sustainable competitiveness. On the one hand, the relationship between employer brand (and attribute congruence) and employee behavior is remote and indirect, which is mediated by their psychological mechanisms, such as satisfaction. In the current context of boundaryless careers, where employees’ careers are becoming more flexible and less dependent on a particular organization [14], an employee who is satisfied with his or her career rather than their limited job is more likely to contribute to proactive behaviors to increase career employability and career thriving. As far as the employees themselves are concerned, satisfied employees have the ability (they have spare resources to craft with because their basic requirements have been met) and motivation (they have a sense of accomplishment for further career development) to take the initiative to innovate [15]. From the point of view of organizational factors, a satisfying career and a supportive organizational environment help individuals to confront and creatively solve problems encountered in their jobs and careers. On the other hand, the aforementioned relationship is ambidextrous, and is dictated by individual traits. For example, the consistent cues of employer brand are notably important for less proactive employees, who lack capability and motivation for information processing.

**Figure 1:**
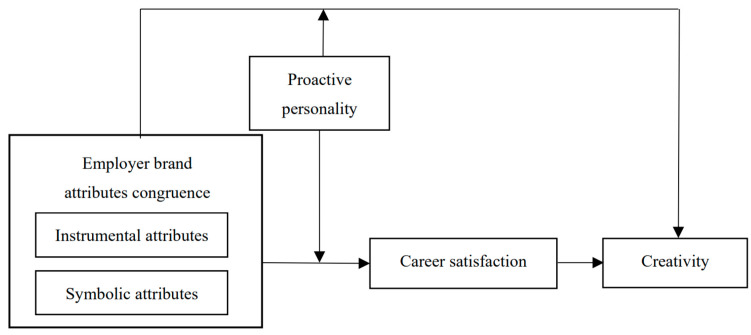
Research model.

Above all, the current study seeks to answer the following specific research questions: (a) what impact does employer brand attributes congruence have on employee creativity, and (b) to what extent does career satisfaction mediate the relationship between employer brand attributes congruence and creativity? In addition, (c) we discuss the boundary condition of proactive personality on the effect of employer brand attributes congruence. Figure 1 displays the research model of this study.

It is hoped that this research will contribute to a deeper understanding of the employer brand and creativity literature. First, this study explores the impact mechanism of employer brand attributes congruence on employee creativity. The perspective of actor–context interaction has attracted much more attention in this field; however, there are strong differences between the contents of the context [16], such as the instrumental and symbolic climate. We go beyond prior studies and seek to examine the joint effect of employer brand attributes on employee creativity. Second, this study sheds light on the important mediation of the individual factors and psychological processes between contextual factors and creativity [17]. We expand on the consequence of employer brand attributes congruence on employees’ satisfaction to their whole career in the current context, which empowers their ability and motivation, and further boosts their creativity. Third, we suggest that a proactive personality is an important motivational variable [18] that determines the intensity and patterns of information processing [8]. Researchers have argued that contextual factors in organizations substantially influence the degree to which employees perform creatively; the impact of these factors and practices may vary as a function of employees’ stable or transient personalities [16,17,19]. In this regard, this paper resolves the question of whether the effect of employer brand attributes congruence is always beneficial or is similar for employees with different levels of proactive personality [20] to some extent.

## 2. Theoretical Background and Hypotheses

### 2.1. Employer Brand Attributes Congruence and Employee Creativity: Mediation of Career Satisfaction

As a part of the broader multidimensional construct of organizational image [21], the employer brand is the image of an organization in the labor market, reflected as the package of functional, economic, and psychological benefits provided by employment, and identified with the employing company [2]. The discussion of the construct and dimensions of employer brand has never stopped, and the most widely accepted classification is an instrumental–symbolic framework. Instrumental attributes describe the job or the organization in terms of objective, concrete, and factual attributes to maximize employees’ benefits and minimize their costs [4], e.g., pay, advancement, job security, and task demands. Employees’ attraction to a company cannot be explained solely based on explicit elements, but also in terms of the symbolic traits and meanings related to person–organization fit, and personality-like terms (e.g., innovativeness, prestige). Symbolic attributes are associated with employees maintaining, enhancing, and expressing their self-image [4]. Since there are dual determining factors in employer brand proposition, an important goal is to evaluate the joint impact of the two dimensions in this regard.

Designing and implementing an HRM system that supports and stimulates employee creative thinking becomes the primary goal for obtaining an organizational competitive advantage, since individual novel ideas can serve as the cornerstone of organizational innovation, transformation, and competition [22]. Creativity refers to the generation of novel and useful ideas concerning products, services, and work methods, which is the most crucial session in all stages of innovation and is the foundation of the process of implementation and assessment [16,23]. However, previous studies have suffered from inadequate theoretical considerations and divergent empirical findings in terms of the effect of employer brand on proactive behaviors, such as creativity [24,25]. As mentioned before, we attribute these issues to the negligence of employer brand attributes congruence.

According to social information processing theory (SIP) [26], the influence and construction of job, task, team, or organizational characteristics, as social information, has an impact on individual creativity through the process of evaluation and choice [27]. On the one hand, the objective characteristics of the task [27] contribute to employee creativity. For instance, modest or relaxed job content and structure [28] allow employees time and energy to generate creative ideas [29]. Similarly, autonomous work [30] urges employees to proactively identify task challenges and explore alternative possibilities through unorthodox approaches, thus promoting set-breaking and experimental behaviors [31]. On the other hand, an organization’s innovative atmosphere, climate, and environment [32,33] encourage employees to work creatively [22]. Overall, both instrumental and symbolic attributes of employer brand are crucial for predicting employee creativity. However, it is not only the content of employer brand that is principal, but also the configuration characteristics of both attributes.

CCT identifies the characteristics of social information. CCT believes that one cue may interact with the effect of the other [34]. Individuals detect cues to make evaluations and tend to maintain consistent patterns among multiple cognitive elements; when the detected cues are consistent, they are more likely to be used jointly in evaluation [35], and the cues will support each other because the latter cue will fit with pre-existing thinking patterns or expectations [36]. However, inconsistent cues will weaken the effectiveness of each other and even cause individuals to focus on the discrepancy or negative cues, which negatively influences individuals’ subsequent evaluations [34,35]. Incongruent information is salient [37] and easy to recall and reconstruct in a retrospective process [8,26], thus some individuals will be distracted by negative cues when multiple cues provide disparate conclusions [35]. That is, inconsistent employer brand attributes may cause individuals to focus on divergent or negative cues, thereby negatively impacting cognitive or decision-making processes.

We believe that consistent contextual cues are particularly critical for regulating employees’ attitudes and behaviors, especially for those who are less proactive. This is because strong instrumental attributes and strong symbolic attributes indicate consistent managerial cues, and employees who are motivated and inspired by an organization’s innovative climate also have access to resources to support their creative thinking as well. In the same vein, the organization will not set inappropriate goals or expectations (weak symbolic attributes) for employees who lack supportive resources (weak instrumental attributes). In contrast, if the supply of resources is scarce but the organization’s innovative climate and culture are strong (weak instrumental attributes–strong symbolic attributes), the employees might be forced to generate creative ideas and have a sense of under-qualification and powerlessness, because the instrumental attributes fail to prompt employees’ innovative ideas. Similarly, if the organization provides employees with sufficient instrumental resources while being weak in creating a creative atmosphere (strong instrumental attributes–weak symbolic attributes), employees will lack the consciousness and motivation needed for creativity. Formally, we summarize this pattern in the following hypothesis:

**H1a.**  *Employer brand attributes congruence is positively related to employee creativity.*

Furthermore, we compare different configurations of instrumental and symbolic attributes along the (in)congruence line. First, we predict that the level of employer brand attributes congruence is positively related to employee creativity, considering that both instrumental and symbolic attributes are boosters of employee creativity. Second, we also postulate that instrumental attributes may play a major role in explaining employees’ career satisfaction and creativity. One reason for this is that current employees put more weight on the expression of instrumental attributes, which means more sufficient and effective information or resources. Another reason is instrumental attributes are more objective, authentic, and verifiable than symbolic ones [34]; the former is reflected in tangible task content, compensation and benefits, etc., which is the ‘inside’ of HRM practice, while the latter is more displayed in the intangible organizational culture or climate, which is the ‘face’ of an organization. Employees are more pragmatic and hold the view of ‘seeing is believing’, so we propose the following hypotheses:

**H1b.**  *Employees will display more creativity in the context of a strong instrumental–strong symbolic employer brand strategy than in the context of a weak instrumental–weak symbolic employer brand strategy.*

**H1c.**  *Employees will display more creativity in the context of a strong instrumental–weak symbolic employer brand strategy than in the context of a weak instrumental–strong symbolic employer brand strategy.*

However, it should be noted that the direct effects of organizational context operate through other unmeasured individual-level variables [32]. We regard career satisfaction as an essential mechanism in the process of work and organizational factors strengthening individual work behaviors under the background of boundaryless careers. Career satisfaction refers to people’s satisfaction with how their career evolves and progresses over time, which is not confined to one job or one organization [38]. The satisfaction of an employee is based on the likelihood that they benefit from invested resources in return [39]. Support from the organization could improve employees’ career satisfaction [40] by strengthening employees’ subjective judgment of demands [41,42], such as compensation, job autonomy, promotion, work–life balance [43,44], etc. Additionally, employees pay more attention to individual value and are much more self-directed when searching for jobs in a boundaryless context; they tend to measure career progress in line with their goals, values, and preferences [20], such as organizational climate, leadership style [41], etc. We regard career satisfaction as the mediator for several reasons: (a) career satisfaction, as an individual emotional judgment, is one of the well-established indicators linking career characteristics and career behaviors [38], (b) it is a key pre-condition of employees’ creative thinking, as one can invest time and energy only if they are satisfied with fundamental demands, (c) and it is a vital concern for managers before paying attention on employees’ motivation and ability to create.

In addition, the congruence of employer brand attributes is quite important when contributing to individuals’ cognition and evaluations [35]. It is not just the content of employer brand attributes that matters, it is the coordination between the two attributes. The two attributes complement and support each other—instrumental attributes are the carrier of symbolic attributes, while symbolic attributes provide a strong atmosphere and perception for instrumental attributes [39]. According to CCT, one cue may interact with and support the effect of the other [34]. The consistency between employer value propositions and the practical supply of demanded resources gives employees faith in not only their current job but also the prospects of their career. The principal reason for this is that a consistent employer brand indicates that the organization does (supports resources) as it declares (claims value and culture), which can enhance the employee’s judgment of their career.

Moreover, proactive behaviors are one of the pivotal consequences that career satisfaction achieves [20]. This paper argues that the improvement of career satisfaction can increase employees’ active innovation behavior for two reasons. First, as far as the employees themselves are concerned, employees who are satisfied with their career development have the ability and motivation to proactively generate and conduct creative ideas [15]. On the one hand, satisfied employees will have a sense of goal achievement and great ease in their careers [45]. Thus, they are able to contribute more to regulate themselves and engage in the organization, owing to sufficient resources (both perceptual and actual resources). On the other hand, satisfied employees are more willing to go further and achieve higher goals due to a sense of accomplishment [46]. For example, they are motivated to perform positively and implement extra-role and discretionary behaviors (such as creativity) beyond work regulations, which is beneficial to the thriving of their career. Second, career satisfaction implies a supportive and encouraging atmosphere in the organization, which is a key factor in improving creativity [47,48,49]. Previous research has also confirmed the relationships between satisfaction and stronger creative performance [49]. Collectively, we propose Hypothesis 2:

**H2.**  *Career satisfaction mediates the relationship between employer brand attributes congruence and employee creativity.*

### 2.2. Employer Brand Attributes Congruence and Employee Creativity: Moderation of Proactive Personality

As explained before, consistent cues are particularly useful for employees with less proactive personalities who rely on external cues for behavioral guidance [9]. By the same token, we argue that the proactive personality is related to information processing patterns. SIP asserts that the method [35] and extensiveness [8] of perception, judgment, and processing are affected by the motivation of information processing [18]. A proactive personality is a work-related and motivational resource, state, or process [50,51] that drives individuals to regulate their surroundings rather than sticking to the status quo and being resigned to life as it is [51,52,53]. Individuals with different levels of proactive personality could process employer brand information heuristically or systematically according to SIP [54]. Systematic processing considers all informational inputs for their relevance, especially their details, for the decision which makes the assessment and scrutinization more comprehensive and analytic; meanwhile, heuristic processing takes a subset of available information into account and focuses on the simple principle to save effort and capacity and formulates the judgments quickly and efficiently [8].

We argue that individuals with lower levels of proactive personality are better at processing congruent employer brand messages according to SIP. The main reason is that less proactive people tend to make less effort when judging and processing social information [8,55]. They would turn to heuristic cues which are easy to access, that is, source characteristics (such as the congruence of multiple messages, credibility, and likability), rather than massage characteristics (e.g., amount, comprehensibility, and the validity of persuasive argumentation) [54]. The cues of congruent employer brand attributes are perceived to be reliable by employees [34], which can be extrapolated to an employer’s trustworthy image. Instead, when cues present inconsistent signals, employees focus on the negative cue and anchor their perceptions of quality accordingly because of the strong salience and diagnosability of negative information [37].

More interestingly, proactive personality or other similar motivations will moderate the negative effects of inconsistent information [8]. On the one hand, SIP labels this desired level of confidence as the sufficiency threshold, which varies according to dispositional factors and individual characteristics. Individuals will continue processing systematically when actual confidence lags back behind the threshold [8]. Proactive individuals hold higher thresholds, so that the gaps between actual confidence and expected confidence divide, which stimulates the potential of employees and promotes their systematic information processing. On the other hand, the discrepancy between employer brand attributes brings about a mismatch in expectations or cognitive schemata [36], which charges more effort from receivers to distinguish the information as well as relieve discomfort [55]. Proactive people are characterized as engaging in situations, showing initiative, and persevering to optimize undesirability context [53,56]; the urgent motivation of continuous improvement [53] helps to promote proactive individuals to actively alleviate and overcome inconsistent employer brand attributes. In addition, proactive individuals are not sensitive to the congruence of employer brand attributes owing to self-serving bias [57]; they attribute career success to personal characteristics [58] rather than their external environment (such as employer brand strategies and other HRM practices). Together, we propose the following hypotheses:

**H3.**  *Proactive personality moderates the direct effect between employer brand attributes congruence and employee creativity. Specifically, low-level proactive personality strengthens the positive impact of employer brand attributes congruence on employee creativity, and high-level proactive personality alleviates the negative impact of employer brand attributes incongruence on employee creativity.*

**H4.**  *Proactive personality moderates the indirect effect between employer brand attributes congruence and employee creativity. Specifically, low-level proactive personality strengthens the positive impact of employer brand attributes congruence on employee creativity via career satisfaction, and high-level proactive personality alleviates the negative impact of employer brand attributes incongruence on employee creativity via career satisfaction.*

## 3. Materials and Methods

### 3.1. Participants and Procedure

To reduce the common method of variance and ensure the validity of the test, this study used paired questionnaires between 100 HR practitioners and employees in the same company, with questionnaires completed by HR practitioners to capture the objective implementation of employer brand and other variables, which were at an individual level completed by employees. The subjects of this study mainly included front-line service employees, such as sales personnel selling products and services from security companies, tellers from commercial banks, and sales agents from insurance companies. Before distributing the questionnaire, the researchers first contacted the subjects to confirm their willingness to participate in the survey, and then the paper questionnaires were handed out and received by mail. The authors asked 100 HR practitioners from 100 financial companies who were willing to fill out a questionnaire for HR, further requiring them to distribute 5 employee questionnaires to other employees (non-HR practitioners) in the same company to complete. Finally, we collected 100 HR questionnaires and 488 employee questionnaires in total, and then we paired these questionnaires by company. The return rate of companies and HR practitioners is 100%, the return rate of employees (non-HR practitioners) is 97.6%. Among the 100 companies surveyed, 77 were from northern China and 23 were from other regions (including eastern China, southern China, Hong Kong, etc.); 35 were banking companies, 25 were security companies, 3 were insurance companies, and 27 were other financial companies; 14 had fewer than 100 employees, 35 had 100–1000 employees, and 51 had more than 1000. The demographic characteristics of the HR managers and subordinates are displayed in Table 1.

### 3.2. Measures

The scales used in the questionnaire survey in this study are all derived from existing research and developed scales (see Appendix A). The responses for all the items were obtained on a five-point Likert scale (1 = completely disagree, 5 = completely agree). According to existing research tests, the validity of each scale was good.

Instrumental employer brand

The scale adopted the scale developed by Lievens et. al. (2003, 2007) [4,59]. The scale includes five dimensions (advancement, pay and benefits, job security, task demands, and flexible working hours) and 15 items: for example, “The company provides diverse career opportunities”, “The company provides training opportunities”, and “Employees have the opportunity to obtain long-term employment in the company”. Cronbach’s α is 0.88.

Symbolic employer brand

For the scale, we adopted the scale developed by Lievens and Highhous (2003) [4]. The scale includes five dimensions (sincerity, innovativeness, competence, prestige, and robustness) and 18 items: for example, “honest”, “sincere”, and “prestigious”. Cronbach’s α is 0.95.

Creativity

We adapted the scale of Tierney, Farmer, and Graen (1999) [60], and retained the original items developed in their pilot study. The scale has 5 items: for example, “Took risks in terms of producing new ideas in doing job”, and “Identified opportunities for new products/processes”. Cronbach’s α is 0.91.

Career satisfaction

For this scale we adopted the scale developed by Greenhaus, Parasuraman, and Wormley (1990) [61]. The scale has 5 items: for example, “I am satisfied with the achievements I have made in my career”, and “I am satisfied with the progress I have made toward meeting my overall career goals”. Cronbach’s α is 0.91.

Proactive personality

This study adopted the shortened version of Bateman and Crant’s (1993) scale [62]. The scale includes 10 items: for example, “I constantly seek new ways to improve life”, and “Wherever I am, I vigorously drive constructive changes within the company”. Cronbach’s α is 0.92.

### 3.3. Analytic Strategy

In this study, we used polynomial regression to test our hypotheses. Previous studies have mainly used different scores or interactions of two variables to test congruency, ignoring the direction of difference, and mixing up diverse configurations of two variables. For example, traditional methods fail to distinguish high–low configurations and low–high configurations. Similarly, polynomial regression is used for testing the relationships between the congruence (i.e., fit, match, similarity, or agreement) between two constructs and their outcomes. Polynomial regression is advantageous when compared to other methods in congruence research that are criticized for methodological problems [63], considering two constructs as well as the joint effects of their interactions, allowing for direct, unqualified, and comprehensive measurements and the interpretation of the two constructs and their covariation [64]. The equation often contains the two constructs and the higher-order (squares) terms. We added Sat (career satisfaction); Ins (instrumental employer brand); Sym (symbolic employer brand); Ins^2^, Sym^2^, Ins × Sym; Ins × Pro (proactive personality); Sym × Pro; Ins^2^ × PP; Sym^2^ × Pro; and Ins × Sym × Pro, which are the interactions with proactive personality, and generated the final equation (control variables are omitted):

(1) $$\begin{array}{c} M_{ij}=b_{0}+b_{1}Ins_{ij}+b_{2}Sym_{ij}+b_{11}In{s_{ij}}^{2}+b_{12}Ins_{ij}\times Sym_{ij}+b_{22}Sy{m_{ij}}^{2}+b_{3}Sat_{\mathrm{ij}}\\ +\sum d_{k}Control_{k\_ij}+e_{ij}+u_{j} \end{array}$$

(2) $$\begin{array}{l} M_{ij}=b_{0}+b_{1}Ins_{ij}+b_{2}Sym_{ij}+b_{11}In{s_{ij}}^{2}+b_{12}Ins_{ij}\times Sym_{ij}+b_{22}Sy{m_{ij}}^{2}+b_{3}P{ro}_{ij}\\ \;\;\;\;\;+b_{13}Ins_{ij}\times P{ro}_{ij}+b_{23}Sym_{ij}\times P{ro}_{ij}+b_{113}In{s_{ij}}^{2}\times P{ro}_{ij}\\ \;\;\;\;\;+b_{123}Ins_{ij}\times Sym_{ij}\times P{ro}_{ij}+b_{223}Sy{m_{ij}}^{2}\times P{ro}_{ij}+\sum d_{k}Control_{k\_ij}+e_{ij}+u_{j} \end{array}$$

in which *i* represents organizations, *j* represents employees in corresponding organization, Equation (1) takes the mediation of career satisfaction into account, and Equation (2) takes the moderation of proactive personality into account. The distribution of congruent and incongruent samples was counted, the samples with instrumental attributes > symbolic attributes accounted for 24.26%, and the samples with instrumental attributes < symbolic attributes accounted for 29.90%, all of which were greater than the threshold of 10%, so it is suitable to for polynomial regression. As suggested by Edwards and Parry, all predictor variables were scale-centered to reduce multicollinearity and facilitate the interpretation of the graphs. This involved subtracting a value of three for the predictors, which were measured on 5-point scales. Finally, we adopted a response surface methodology (RSM, the program can be downloaded from https://tarheels.live/jeffreyedwardswebsite/home/downloads/ (accessed on 22 May 2024)) to display the results of the polynomial regression [63,65] and used block variables [66] to examine the indirect effect.

## 4. Results

Table 2 shows the means, standard deviations, and intercorrelations of the variables.

Table 3 shows the results of the model fit comparisons. The results of the confirmatory factor analyses show that the hypothesized five-factor model has a satisfactory fit (*χ*^2^/*df* = 3.44, RMSEA = 0.07, CFI = 0.91, TLI = 0.90, SRMR = 0.04) and has a significantly better fit than all of the alternative models.

### 4.1. Effect of Employer Brand Attributes Congruence on Employee Creativity via Career Satisfaction

**Figure 2:**
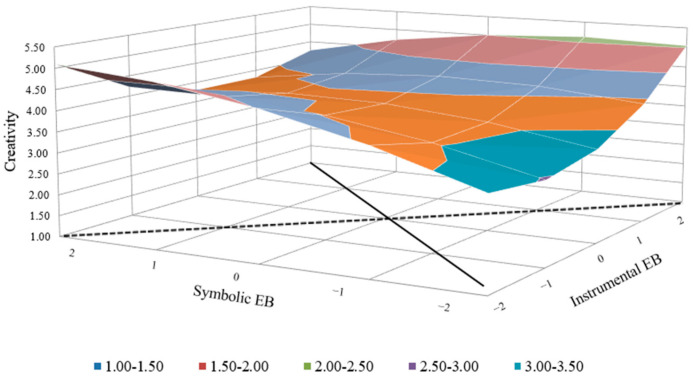
Influence of employer brand attributes congruence on creativity.

The regression results given in Table 4 show that after the squared term is added (model 4), the model is significant (F = 3.17, *p* < 0.01), and Δ*R*^2^ is significant as well, indicating that it is better to analyze the results using congruence (Edwards, 2002 [65]). According to the polynomial regression and response surface analysis (Figure 2), employer brand attributes congruence has commensurate compatibility and a curvilinear-level effect with a contingency on employee creativity (*a*1 ≠ 0, *a*2 ≠ 0, *p*10 ≠ 0, *a*4 ≠ 0 and *p*11 ≠ 1) (*a*1 = the slope of the surface along the fit line, *a*2 = the curvature of the surface along the fit line, *a*3 = the slope of the surface along the misfit line, *a*4 = the curvature of the surface along the misfit line, LSQ = lateral shift and lateral shift quantity along the misfit line, *p*10 = the intercept of the first principal axis, and *p*11 = the slope of the first principal axis.) [67]. The slope and curvature along the congruence line (slope = *b*1 + *b*2 = 0.28, *p* < 0.1; curvature = *b*3 + *b*4 + *b*5 = −0.04, *p* > 0.05) reveal that there is a slightly upward tendency of the congruence line and a strong instrumental–strong symbolic employer brand strategy that is correlated to a higher level of employee creativity than a weak instrumental–weak symbolic employer brand strategy. The slope and curvature along the incongruence line (slope = *b*1 − *b*2 = −0.02, *p* > 0.05; curvature = *b*3 − *b*4 + *b*5 = 0.30, *p* > 0.05) and positive LSQ (lateral shift quantity, (*b*2 − *b*1)/[2(*b*3 − *b*4 + *b*5)]) suggest that a weak instrumental–strong symbolic employer brand strategy is correlated to a slightly higher (nonsignificant) level of employee creativity than a strong instrumental–weak symbolic employer brand strategy. Hypotheses 1a and 1b are supported, while Hypothesis 1c was not supported.

As can be seen, model 3 added career satisfaction as a mediator and the regression coefficient of career satisfaction is significant (B = 0.45, *p* < 0.001), and so is the Δ*R*^2^. Following Edwards and Cable (2009) [66], we constructed block variables representing employer brand attributes congruence to test its indirect effect. The results show that the block variable for employer brand attributes congruence is positively related to career satisfaction (*a* = 0.98, [0.42, 1.53]), and career satisfaction is positively associated with employee creativity (*b* = 0.45, [0.37, 0.52]). Bias-corrected bootstrap confidence intervals of the indirect effect (*ab*) of employer brand attributes congruence (block variable) on employee creativity (*ab* = 0.43, [0.17, 0.73]) are inclusive or exclusive of zero, while the direct effect of block variable on creativity is insignificant (effect size = 0.17, [−0.25, 0.58]). Overall, these findings support Hypothesis 2; the relationship of employer brand attributes congruence on creativity is fully mediated by career satisfaction.

### 4.2. Moderating Role of Proactive Personality

As shown in Table 5, the current study conducts the polynomial regression equation with the moderator variable and its interactions. After controlling other variables, model 1 adds interactions and sees an increase in *R*^2^ (*p* < 0.001), and the coefficient of proactive personality is significant (B = 0.70, *p* < 0.001); model 2 takes employee creativity as the dependent variable, and the coefficient of proactive personality is significant (B = 0.60, *p* < 0.001). Although bias-corrected bootstrap confidence intervals of the indirect effect of the conditional effect of interactive variables on employee creativity are inclusive of zero, the Z-tests of the conditional effects show that the difference in effect sizes between high-level proactive personality and low-level proactive personality is significant (direct effect, Z = 21.82; indirect effect, Z = 1.70), which means there are notable differences between the two groups.

To further analyze the moderating mechanism of proactive personality and the moderated mediation, this study divides the sample into two subsamples based on the median of the moderating variable, and then carries out a polynomial regression (Table 6) and response surface analysis on the two subsamples.

**Figure 3:**
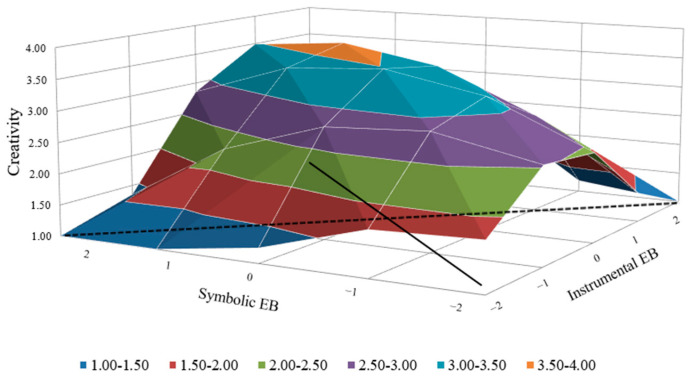
Influence of employer brand attributes congruence on creativity (low-level proactive personality).

**Figure 4:**
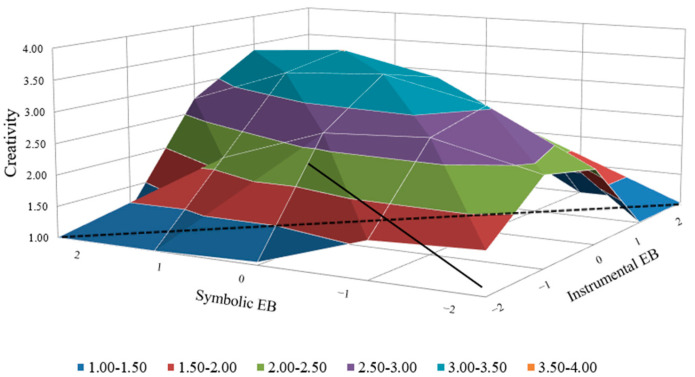
Influence of employer brand attributes congruence on creativity via career satisfaction (low-level proactive personality).

As Figure 3 shows, for less proactive employees, the curve trends significantly downward along the incongruence line (*a*2 = −0.24, *p* < 0.05), and the slope is significantly positive along the congruence line (*a*1 = 0.37, *p* < 0.05). More importantly, the slope and curvature along the congruence line of the relationship between employer brand attributes congruence and employee creativity via career satisfaction (*a*1 = 0.36, *p* < 0.05; *a*2 = −0.26, *p* < 0.05) reports a downward tendency along the congruence line (as shown in Figure 4). There is an increase in employee creativity associated with a strong instrumental–strong symbolic brand strategy rather than a weak instrumental–weak symbolic employer brand strategy.

**Figure 5:**
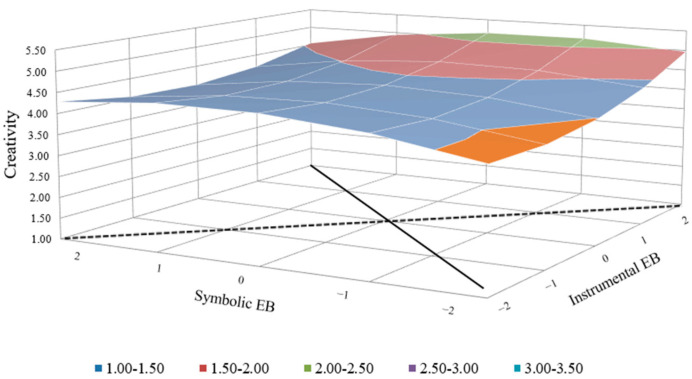
Influence of employer brand attributes congruence on creativity (high-level proactive personality).

**Figure 6:**
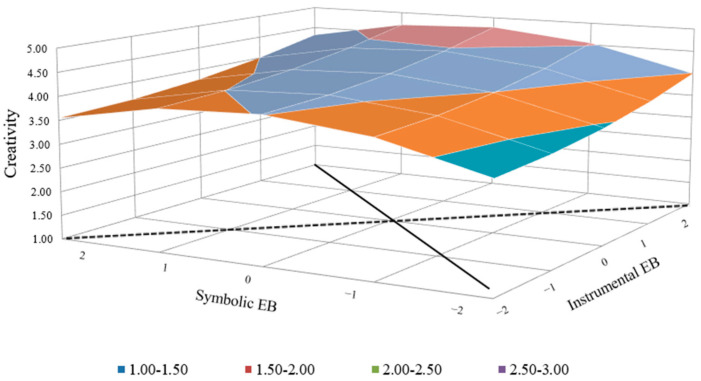
Influence of employer brand attributes congruence on creativity via career satisfaction (high-level proactive personality).

Although, for highly proactive employees, the slope and curvature of employer brand attributes congruence and employee creativity are insignificant, neither are those of employer brand attributes congruence or employee creativity via career satisfaction, as displayed by Figure 5 and Figure 6, which means proactive personality can restrain the negative effect of incongruence; therefore, Hypothesis 3 and Hypothesis 4 are supported.

## 5. Discussion

### 5.1. Theoretical Implications

By and large, the present study contributes to the employer brand and creativity literature in at least three ways. First, it contributes to the growing, but still quite sparse, empirical literature adopting an interactional approach to creativity by investigating the effect of employer brand and its synergistic function with individual traits. We expand the influence of employer brand, not only to the recruitment in the early stage of employment, but also to implement the role of stimulating the initiative behaviors of current employees in the post-employment process. While the relationship between employer brand and creativity is quite circumstantial, we take career satisfaction as a fundamental psychological mechanism in the process. The content and configuration of employer brand strengthen employees’ satisfaction, with not only the particular organization but also their whole career, thus further boosting their initiative behaviors (such as creativity) to take their career to the next level. Furthermore, Zhou (2003) [19] pointed out that work is much needed to identify the full range of conditions that might suppress or support creativity for individuals with more or less proactivity. The goal of this current research is to find ways to promote creativity among employees with different levels of proactive personality; the findings of our empirical study provide further evidence that the information processing patterns to different employer brand configurations vary from employees with highly proactive personality to those with less proactive personality.

Second, the present study appears to be the first attempt to thoroughly examine the congruence of employer brand attributes. The conclusions also support the notion that synergistic HRM practices combine and complement others; HRM bundles consisting of multiple complementary practices are typically considered superior to individual best practices in influencing performance [68]. This study confirms the synergistic effect of job/organization factors and the characteristics of organizations [4]. Further, this paper integrates, verifies, and expands the CCT and SIP. Situational factors do not work similarly for all individuals [38,52,53], and we go beyond previous research and discuss the associations between (in)congruence and proactive personality. Additionally, social information has numerous and varied operational definitions, whereas little is known about the job and task environment characteristics in the SIP model [69]. This study takes the instrumental and symbolic attributes of the employer brand into consideration simultaneously, which complement and consummate SIP.

Last, the current study indicates that a proactive personality is a crucial boundary condition in which employee creativity is improved. This study confirms that for less proactive individuals, instrumental and symbolic attributes are complementary and could reinforce each other; when either of the two cues provides a weak or negative signal, regardless of the strength of the positive cues, the overall evaluations are decreased [37]. Meanwhile, individuals with a high level of proactive personality are better at comprehensively examining all information inputs, and can adopt inconsistent information more efficiently than less proactive people, thereby promoting career satisfaction and creative behavior in their own career development. Thus, high levels of proactive personality can compensate for the negative effects of the incongruence of employer brand attributes because they are prone to making more effort when processing information. The main reasons for this are that (a) a highly proactive personality means more gaps between actual confidence and the confident threshold, and (b) proactive individuals are less sensitive to external cues, while they are more than equal in regulating their environment.

### 5.2. Practical Implications

The findings of this study have several practical implications. Most of all, organizations should consider them rigorously when shaping employer brand strategies and make the best of the situation according to the personalities of employees as well. There are two possible ways to promote employee creativity in general. In particular, on the one hand, the organization needs to meet the pragmatic experience and demands of employees, such as comprehensive training and learning activities, promotion channels, fair and reasonable compensation and benefits, etc. On the other hand, it is also vital to enhance the strength of symbolic employer brand expression, and promote the organization’s related culture or climate to improve the perceived fit between employees and the organization. In turn, this increases employees’ career satisfaction and ultimately strengthens their work behavior. More importantly, the congruence of these two aspects cannot be neglected when designing, implementing, and disseminating employer brand strategy. Moreover, considering the essential mediating mechanism of career satisfaction in the context of boundaryless careers, organizations have to give prominence to the long-term career development of employees, offer them more sustainable resources like learning and training approaches, and foster everlasting values that fit their particular career.

Further, according to our findings, managers should propose employee value propositions to subordinates on a case-by-case basis. Specifically, for less proactive employees, the organization should strive to convey the connotation of the employer brand with consistency in the two dimensions to reduce their psychological insecurity, anxiety, and dissonance, improve career satisfaction, and support them to generate, transmit, and implement creative thinking. One sincere piece of advice is to act on what you say, or, if you are not able to provide necessary resources or support, stay low-profile rather than boasting about symbolic attributes. For employees with high proactivity who excel at utilizing resources to control and change inconsistent contexts, organizations should provide more support (either ample resources or a strong atmosphere) even though this is imbalanced. Proactive employees are likely to consider extra information an opportunity; in this circumstance, the best level of employee creativity does not necessarily appear when congruence, or, on the contrary, slight incongruence, is more likely to promote employee creativity [67].

Last, although it does not correspond with our hypothesis, the results show that the weak instrumental–strong symbolic employer brand strategy is correlated to a slightly higher level of employee creativity than the strong instrumental–weak symbolic employer brand strategy among all the samples. One explanation for this is that symbolic attributes are more relevant [9] and crucial to employee creativity. Organizations stimulate employee creativity in vain when they provide resources without developing their awareness of creative thinking. In other words, it is internal inspiration that counts when boosting employee creativity. Thus, we call for placing a premium on symbolic emphasis, especially when promoting employees’ creativity and innovation.

### 5.3. Limitations and Future Research

This study provides the first investigation into the influence mechanism of employer brand attributes congruence on employee attitudes and behaviors. It has certain limitations that need to be acknowledged. First, there was no objective measure of creativity available on our research site with which we could explore convergence with the self-reported ratings. It will be more informative to use multiple indices to tap into the different aspects of employee creativity (e.g., self-reported ratings, supervisor ratings, and objective measures, such as the number of patents) [70]. Moreover, the ultimate goal of improving working patterns and thinking creatively is to promote career success [71], and future research could allow for further investigation into the relationship between employer brand and career success via creativity. Second, this study only examines the impact of employer brand on career satisfaction and the creativity of current employees, without taking potential applicants into account. In this regard, future research can consider examining the differences in the efficiency of instrumental and symbolic employer brand among different groups using experimental research. Last of all, a proactive personality exerts beneficial lagged effects on increases in organizational resources and job characteristics, as well as on decreases in organizational constraints [72]. However, there are very few published longitudinal investigations on different job-seeking stages [73]. In addition, this is so even when paired questionnaires give us some confidence that the assumptions about causality implied by our analysis would not be unreasonable here. Longitudinal studies or experiments should be designed to capture the lagged variances in variables, enhance causal inference, and track the evidence of employer-related decisions and behaviors relating to potential applicants or current employees in the future [74].

## Figures and Tables

**Table 1:** Sample demographic characteristics.

Variables	Characteristics	Number		Frequency	
		HR Practitioners		Employees	
Gender	Male	48	48.0%	231	47.3%
	Female	52	52.0%	257	52.7%
Age	Before 1960	1	1.0%	3	0.6%
	1960–1969	4	4.0%	29	5.9%
	1970–1979	17	17.0%	62	12.7%
	1980–1989	64	64.0%	285	58.4%
	After 1990	14	14.0%	109	22.3%
Tenure	Less than 1 year	0	0.0%	19	3.9%
	1–3 years	11	11.0%	58	11.9%
	4–5 years	15	15.0%	78	16.0%
	6–10 years	35	35.0%	170	34.8%
	More than 11 years	39	39.0%	163	33.4%
Organizational level	Company’s headquarters	47	47.0%	220	45.1%
	Second-level institutions	37	37.0%	157	32.2%
	Third-level institutions	11	11.0%	61	12.5%
	Other levels	5	5.0%	50	10.2%
Position	Senior managers	6	6.0%	24	4.9%
	Middle managers	34	34.0%	81	16.6%
	Grassroots managers	27	27.0%	126	25.8%
	Grassroots employees	33	33.0%	257	52.6%

**Table 2:** Means, SDs, and correlations.

Variables	Mean	SD	1	2	3	4	5
1. Instrumental attributes	3.62	0.59	1				
2. Symbolic attributes	3.86	0.69	0.63 ***	1			
3. Career satisfaction	3.33	0.85	0.07	0.09	1		
4. Proactive personality	3.69	0.68	0.04	0.05	0.59 ***	1	
5. Creativity	3.73	0.74	0.11 *	0.05	0.50 ***	0.70 ***	1

Note: * *p* < 0.05, *** *p* < 0.001.

**Table 3:** Confirmatory factor analysis.

Variables	*χ*^2^/*df*	RMSEA	CFI	TLI	SRMR
5-factor model (Ins; Sym; Sat; Pro; Cre)	3.44	0.07	0.91	0.90	0.04
4-factor model (Ins + Sym; Sat; Pro; Cre)	4.88	0.09	0.85	0.84	0.06
3-factor model (Ins + Sym; Sat + Pro; Cre)	6.76	0.11	0.78	0.76	0.08
2-factor model (Ins + Sym + Sat + Pro; Cre)	8.28	0.12	0.72	0.69	0.08
1-factor model (Ins + Sym + Sat + Pro + Cre)	13.93	0.17	0.50	0.46	0.16

Note: Ins is instrumental attributes, Sym is symbolic attributes, Sat is career satisfaction, Pro is proactive personality, and Cre is employee creativity.

**Table 4:** Polynomial regression of employer brand attributes congruence on creativity.

Variables	Career Satisfaction				Creativity							
	Model 1		Model 2		Model 3		Model 4		Model 5		Model 6	
	B	SE	B	SE	B	SE	B	SE	B	SE	B	SE
Intercept	3.36 ***	0.28	3.29 ***	0.28	3.93 ***	0.24	3.86 ***	0.24	3.75 ***	0.21	2.43	0.24
Gender	−0.05	0.09	−0.05	0.09	−0.19 **	0.07	−0.19 **	0.07	−0.17 **	0.06	−0.17 +	0.06
Age	0.09	0.06	0.09	0.06	0.07	0.05	0.07	0.05	0.02	0.04	0.03	0.04
Organization Level	0.11 *	0.04	0.10 *	0.4	0.06	0.04	0.06	0.04	0.01	0.03	0.01	0.03
Position	−0.15 **	0.05	−0.16 **	0.05	−0.09 *	0.04	−0.10 *	0.04	−0.03	0.04	−0.02	0.04
Instrumental Attributes			0.07	0.17			0.13	0.15	0.11	0.13		
Symbolic Attributes			−0.08	0.17			0.15	0.15	0.17	0.13		
Ins^2^			0.48 **	0.17			0.22	0.15	0.01	0.13		
Ins × Sym			−0.53 +	0.28			−0.17	0.24	0.05	0.20		
Sym^2^			0.19	0.14			−0.09	0.12	−0.17	0.11		
Career Satisfaction									0.45 ***	0.04	0.44 ***	0.04
Block Variable											0.17	0.21
F	3.98 **		3.20 **		3.67 **		3.17 **		18.34 ***		28.44 ***	
R^2^	0.04		0.07		0.04		0.07		0.32		0.30	
ΔR^2^	0.04 **		0.03 **		0.04 **		0.03 **		0.25 ***		0.30 ***	
Congruence (Ins = Sym)												
Slope (*b*1 + *b*2)							0.28 +		0.28 *			
Curvature (*b*3 + *b*4 + *b*5)							−0.04		−0.11			
Incongruency (Ins = −Sym)												
Slope (*b*1 − *b*2)							−0.02		−0.06			
Curvature(*b*3 − *b*4 + *b*5)							0.30		−0.21			

Notes: + *p* < 0.1, * *p* < 0.05, ** *p* < 0.01, and *** *p* < 0.001. Ins is instrumental attributes, Sym is symbolic attributes, Sat is career satisfaction, Pro is proactive personality, and Cre is employee creativity.

**Table 5:** Moderation of proactive personality.

Variables	Career Satisfaction		Creativity			
	Model 1		Model 2		Model 3	
	B	SE	B	SE	B	SE
Intercept	3.47 ***	0.18	3.48 ***	0.18	3.95 ***	0.17
Gender	−0.16 **	0.05	−0.15 **	0.05	−0.15 **	0.05
Age	0.01	0.04	0.00	0.04	0.01	0.04
Organization Level	0.01	0.03	0.00	0.03	0.01	0.03
Position	−0.03	0.03	−0.02	0.03	−0.01	0.03
Instrumental Attributes	0.02	0.16	0.02	0.16		
Symbolic Attributes	0.22	0.16	0.22	0.16		
Ins^2^	−0.32 +	0.19	−0.34 +	0.18		
Ins × Sym	0.50 +	0.29	0.52 +	0.28		
Sym^2^	−0.31 *	0.14	−0.32 *	0.14		
Proactive Personality	0.70 ***	0.08	0.60 **	9.08	0.64 ***	0.05
Ins × Pro	0.26 +	0.15	0.24	0.15		
Sym × Pro	−0.22	0.17	−0.20	0.16		
Ins^2^ × Pro	0.25	0.17	0.25	0.16		
Ins × Sym × Pro	−0.50 +	0.27	−0.48 +	0.27		
Ins^2^ × Pro	0.25 +	0.13	0.23 +	0.13		
Career Satisfaction			0.15 ***	0.04	0.15 ***	0.04
Block Variable					−0.12	0.19
Block Variable × Pro					0.29	0.27
Sat × Pro					−0.01	0.04
F	29.04 ***		29.25 ***		48.84 ***	
R^2^	0.54		0.55		0.53	
ΔR^2^	0.47 ***		0.02 ***		0.53 ***	

Notes: + *p* < 0.1, * *p* < 0.05, ** *p* < 0.01, and *** *p* < 0.001. Ins is instrumental attributes, Sym is symbolic attributes, Sat is career satisfaction, Pro is proactive personality, and Cre is employee creativity.

**Table 6:** Moderated mediation.

Variables	Creativity							
	Low-Level Pro				High-Level Pro			
	Model 1		Model 2		Model 3		Model 4	
	B	SE	B	SE	B	SE	B	SE
Intercept	3.49 ***	0.29	3.41 ***	0.27	4.39 ***	0.31	4.23 ***	0.29
Gender	−0.07	0.09	−0.07	0.08	−0.24 **	0.09	−0.24 **	0.09
Age	0.03	0.06	0.03	0.06	0.03	0.06	−0.00	0.06
Organization Level	−0.03	0.05	−0.03	0.04	0.07	0.05	0.03	0.04
Position	−0.04	0.05	−0.01	0.05	−0.06	0.05	−0.02	0.05
Instrumental Attributes	0.27	0.22	0.22	0.20	0.18	0.17	0.19	0.16
Symbolic Attributes	0.10	0.20	0.14	0.19	0.01	0.18	0.08	0.17
Ins^2^	−0.43 +	0.24	−0.48 *	0.22	0.10	0.16	0.04	0.15
Ins × Sym	0.40	0.34	0.46	0.32	−0.06	0.27	−0.00	0.25
Sym^2^	−0.22	0.17	−0.24	0.16	−0.10	0.14	−0.16	0.13
Career Satisfaction			0.32 ***	0.05			0.32 ***	0.06
F	1.26		4.87 ***		1.78		5.00 ***	
R^2^	0.06		0.20		0.08		0.22	
ΔR^2^	0.06		0.16		0.08		0.14	
Congruence (Ins = Sym)								
Slope (*b*1 + *b*2)	0.37 *		0.36 *		0.27		0.18	
Curvature (*b*3 + *b*4 + *b*5)	−0.24 *		−0.26 *		−0.12		−0.05	
Incongruency (Ins = −Sym)								
Slope (*b*1 − *b*2)	0.17		0.08		0.11		0.17	
Curvature (*b*3 − *b*4 + *b*5)	−1.04		−1.18		−0.11		0.06	

Notes: + *p* < 0.1, * *p* < 0.05, ** *p* < 0.01, and *** *p* < 0.001. Ins is instrumental attributes, Sym is symbolic attributes, Sat is career satisfaction, Pro is proactive personality, and Cre is employee creativity.

## Data Availability

The raw data supporting the conclusions of this article will be made available by the authors upon request.

## Appendix A

Instrumental Employer Brand Items

“Please indicate the extent to which you agree that each statement describes job/organizational attributes of your company.”

1. The company offers diverse career opportunities.
2. The company offers prospects for higher positions.
3. The company offers the possibility to build a career.
4. This company has a good benefit package.
5. The company offers the possibility to make a lot of money.
6. In general, the wages in the company are high.
7. The company offers the possibility to hold a permanent position.
8. The company offers job security.
9. The company offers people a job for life.
10. The company offers prospects for a certain future.
11. The company offers the possibility to practice a diverse range of jobs.
12. The company offers the possibility to choose from a diversity of jobs.
13. Working in the company offers a lot of variety.
14. The company offers a wide range of jobs.
15. This company has flexible work hours.

Symbolic Employer Brand Items

“Please indicate the extent to which you agree that each statement describes trait inferences of your company.”

1. Honest.
2. Sincere.
3. Cheerful.
4. Friendly.
5. Daring.
6. Trendy.
7. Exciting.
8. Cool.
9. Spirited.
10. Young.
11. Secure.
12. Intelligent.
13. Reliable.
14. Upper class.
15. Prestigious.
16. Masculine.
17. Strong.
18. Robust.

Career Satisfaction Items

“Please indicate the extent to which you agree that each statement currently describes yourself.”

1. I am satisfied with the success I have achieved in my career.
2. I am satisfied with the progress I have made toward meeting my overall career goals.
3. I am satisfied with the progress I have made toward meeting my goals for income.
4. I am satisfied with the progress I have made toward meeting my goals for advancement.
5. I am satisfied with the progress I have made toward meeting my goals for the development of new skills.

Creativity Items

“Please indicate how often the following statements characterize you.”

1. Took risks in terms of producing new ideas in doing job.
2. Identified opportunities for new products/processes.
3. Generated novel, but operable work-related ideas.
4. Served as a good role model for creativity.
5. Generated ideas revolutionary to our field.

Proactive Personality Items

“Please indicate the extent to which you agree that each statement currently describes yourself.”

1. I constantly seek new ways to improve life.
2. Wherever I am, I vigorously drive constructive changes within the company.
3. The most exciting thing for me is to see my ideas come to life.
4. If I encounter something I don’t like, I find ways to address it.
5. Regardless of the size of the opportunity for success, as long as I believe in something, I make it a reality.
6. Even in the face of opposition, I am willing to persist with my ideas.
7. I am adept at spotting opportunities.
8. I am always looking for better ways to do things.
9. If I believe in an idea, no difficulty can stop me from realizing it.
10. I can identify good opportunities earlier than others.
